# Average trapping time on weighted directed Koch network

**DOI:** 10.1038/s41598-019-51229-2

**Published:** 2019-10-10

**Authors:** Zikai Wu, Yu Gao

**Affiliations:** Business School, University of Shanghai for Science and Technology, Shanghai, 200093 China

**Keywords:** Applied mathematics, Complex networks

## Abstract

Numerous recent studies have focused on random walks on undirected binary scale-free networks. However, random walks with a given target node on weighted directed networks remain less understood. In this paper, we first introduce directed weighted Koch networks, in which any pair of nodes is linked by two edges with opposite directions, and weights of edges are controlled by a parameter *θ* . Then, to evaluate the transportation efficiency of random walk, we derive an exact solution for the average trapping time (ATT), which agrees well with the corresponding numerical solution. We show that leading behaviour of ATT is function of the weight parameter *θ* and that the ATT can grow sub-linearly, linearly and super-linearly with varying *θ* . Finally, we introduce a delay parameter *p* to modify the transition probability of random walk, and provide a closed-form solution for ATT, which still coincides with numerical solution. We show that in the closed-form solution, the delay parameter *p* can change the coefficient of ATT, but cannot change the leading behavior. We also show that desired ATT or trapping efficiency can be obtained by setting appropriate weight parameter and delay parameter simultaneously. Thereby, this work advance the understanding of random walks on directed weighted scale-free networks.

## Introduction

In recent years, complex networks have received attention from numerous researchers^1^. As a fundamental tool developed to describe the dynamic process of networks, random walks on a graph has been widely employed to evaluate dynamical properties of different complex networks^2^, such as chemical kinetics in molecular systems^3^, page search in the World Wide Web^4^, and lighting harvesting in antenna systems^5,6^. In addition to the applications in network science^7–9^, random walks has also been applied to other subjects (e.g., image segmentation^10^, the normalized Laplacian spectrum of a graph^11,12^ and signal propagation in proteins^13^).

One of the main foci in studies of random networks is the mean first passage time (MFPT)^14–18^, which has been defined as the expected steps that a walker starting in node *i* will require to reach node *j* for the first time^19^. When the target node *j* is given or assumed as the trap node, the MFPT is regarded as the mean first passage time to the trap node, which is also called the average trapping time (ATT). The ATT for a given trap has been used as a quantitative indicator of trapping efficiency to evaluate the process of random walks^16,20,21^. Researchers have studied the construction and dynamic process of differing undirected fractal networks, such as T-fractals^22–24^, Sierpinski fractals^7,25,26^, and Caley trees^27–30^ and other structures^31–33^.

Previous works have provided the framework for calculating ATT on different undirected scale-free networks. However, many networks in real life are directed and the directed edges linking pairs of nodes may have considerable influences on dynamic processes^34,35^. In comparison, the understanding of random walks process in directed weighted networks is still inadequate. In particular, for random walks in fractal networks or non-fractal networks^36,37^, a significant issue is to explore the effect of weighted and directed edges on the average trapping time (ATT) with a given target node. Although some efforts have been made with several weighted and directed models^38–41^, additional work is needed to deepen the understanding of the impact of the weight and direction on random walks. Below, we first propose a weighted directed Koch network^42–45^, where each pair of nodes has two edges with opposite directions^46^, and the weights of edges are controlled by a weight parameter *θ*. Then, building on the time-delay system theory^47–49^, we further introduce a delay parameter *p* into the weighted directed network.

Following the introduction of weighted directed Koch network, we derive the exact analytical expression of ATT for a given target node. We find that as *θ* goes from 0 to infinity, the ATT of the target grows sublinearly, linearly, and surperlinearly depending on the value of the weight parameter. We also calculate the ATT on weighted directed Koch network with a delay parameter. The result shows that delay parameter can only change the coefficient of the ATT, but leaves the leading scale unchanged. In summary, the results advance our understanding of random walks process in directed weighted Koch network.

## Results

### Construction and properties of directed weighted Koch network

**Figure 1:**
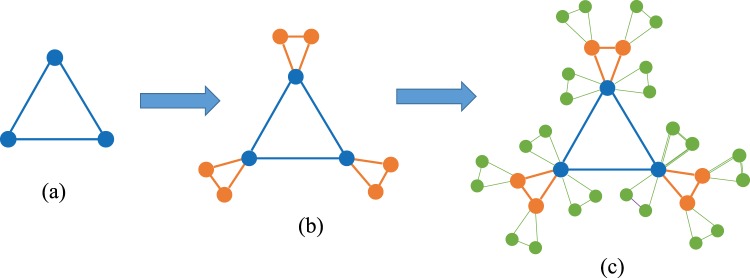
The first three generations of Koch network. (**a**–**c**) are the first three generations of Koch network,respectively. The blue, yellow and green nodes are represent the generations 0,1, and 2, respectively.

The Koch network is derived from the Koch curve, a well-known fractal, in which each node in the network corresponds to one triangle in the curve. The generation procedure of binary Koch networks was detailed in^44^. Let K(g) denotes the network after *g* iterations. For *g* = 0, K(0) is a triangle containing three nodes. For *g* ≥ 1, K(g) can be obtained from K(g^−1^) as follows: For each existing node, two new nodes are created and attached to the existing node to form a triangle^44^. Figure 1 shows the construction process of the first three generations. We note that L(g), the number of triangles in K(g), equals to 4^*g*^. The variable *L*_*v*_(*g*), the number of nodes created at g *th* generation, equal to 6 × 4^*g*−1^. The variable *L*_*e*_(*g*), the number of edges created at g *th* generation, equal to 9 × 4^*g*−1^. Besides, the variable *N*_*g*_, the number of the nodes in K(g), equals to 2 × 4^*g*^ + 1^44^.

Based on the undirected and unbiased Koch network, we propose a weighted directed Koch network by introducing a weight parameter *θ* quantifying the reciprocating relation between edges. In other words, the edge in the binary network is replaced by two opposite directed edges with different weights.

**Figure 2:**
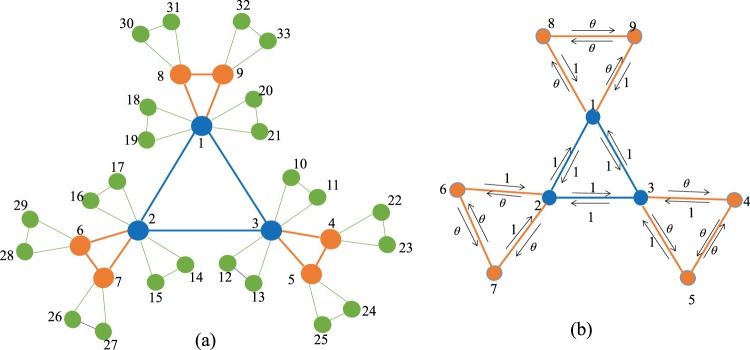
The weighted directed Koch network construction. (**a**) is the labels of nodes for unweighted and undirected Koch model, (**b**) is an example of directed weighted edges’ construction between node 1 and its neighbors for the first two steps.

Let $\overrightarrow{K}(g)$ represent the weighted directed Koch network at generation *g* and *W*_*ij*_(*g*) denote the weight of the edge linking node *i* and node *j* in $\overrightarrow{K}(g)$. Then we define *W*_*ij*_(*g*) > 0, if node *i* and node *j* are adjacent, and *W*_*ij*_(*g*) = 0, otherwise. The weights of edges in the network are defined recursively as follows. When *g* = 0, $\overrightarrow{K}\mathrm{(0)}$ is a triangle containing three nodes, in which *W*_1,2_(0) = *W*_2,1_(0) = *W*_2,3_(0) = *W*_3,2_(0) = *W*_1,3_(0) = *W*_3,1_(0) = 1. When *g* = 1, each node in $\overrightarrow{K}\mathrm{(0)}$ is treated as the mother node and produce two new nodes, which and mother node are connected each other to form a new triangle. In the new triangle, the weight of the two edges linking mother node and new produced nodes is *θ* times that of edges linking mother node and its old neighbors, and the weight of edges linking new nodes and mother node equals to that of edges linking mother node’s old neighbors and mother node. As a result, *W*_1,8_(1) = *θW*_1,3_(0), *W*_8,1_(1) = *W*_3,1_(0), *W*_1,9_(1) = *θW*_1,2_(0), *W*_9,1_(1) = *W*_2,1_(0), *W*_8,9_(1) = *θW*_8,1_(1), *W*_9,8_(1) = *θW*_9,1_(1), *W*_2,6_(1) = *θW*_2,3_(0), *W*_6,2_(1) = *W*_3,2_(0), *W*_2,7_(1) = *θW*_2,1_(0), *W*_7,2_(1) = *W*_1,2_(0), *W*_6,7_(1) = *θW*_6,2_(1), *W*_7,6_(1) = *θW*_7,2_(1), *W*_3,4_(1) = *θW*_3,2_(0), *W*_4,3_(1) = *W*_2,3_(0), *W*_3,5_(1) = *θW*_3,1_(0), *W*_5,3_(1) = *W*_1,3_(0), *W*_4,5_(1) = *θW*_4,3_(1), *W*_5,4_(1) = *θW*_5,3_(1). When *g* > 1, each node of each triangle in $\overrightarrow{K}(g-\mathrm{1)}$ generates one triangle respectively, and the iteration rules governing the weight of edges in new produced triangles are the same as those mentioned above. Figure 2 shows the succession process of the reciprocity weights.

In the directed weighted network, we define the out-strength and in-strength of node *i* at generation *g* as ${S}_{i}^{+}(g)={\sum }_{j\mathrm{=1}}^{{N}_{g}}{W}_{ij}(g),{S}_{i}^{-}(g)={\sum }_{j\mathrm{=1}}^{{N}_{g}}{W}_{ji}(g)$ respectively. Based on the evolution rule of network, it can be shown that ${S}_{i}^{+}(g\mathrm{)=2(}\theta +{\mathrm{1)}}^{g-{g}_{i}}$, where *g*_*i*_ represents the generation at which the node *i* is created.

### Numerical formulations of ATT in directed weighted Koch network

In this paper, we address a special biased random walks with a given target node. For convenience, we label all the nodes in the following way: the initial three nodes in $\overrightarrow{K}\mathrm{(0)}$ are labeled as 1, 2 and 3, respectively. Let *N*_*g*−1_ + 1, *N*_*g*−1_ + 2, …, *N*_*g*_ denote the new nodes created at *g* th generation in $\overrightarrow{K}(g)$, which are shown in Fig. 2. We assume that the target node is placed at any node in K(0). Without loss of generality, let node 1 be target node. This stochastic process of random walks is characterized by the transition matrix *P*_*g*_. The element of *P*_*g*_ named *p*_*ij*_(*g*) denotes the transition probability of jumping from *i* to *j* in each time step. It satisfies ${\sum }_{j\mathrm{=1}}^{{N}_{g}}\,{p}_{ij}(g\mathrm{)=1}$.

Let ${T}_{i}^{(g)}$ denote the mean first passage time for a walker moving from node *i* to target node in $\overrightarrow{K}(g)$. Then, it obeys the equation

1 $$\begin{array}{rcl}{T}_{i}^{(g)} & = & \mathop{\sum }\limits_{j\mathrm{=1}}^{{N}_{g}}\,{p}_{ij}(g)({T}_{j}^{(g)}+\mathrm{1)}\\  & = & \mathop{\sum }\limits_{j\mathrm{=1}}^{{N}_{g}}{p}_{ij}(g){T}_{j}^{(g)}+\mathop{\sum }\limits_{j\mathrm{=1}}^{{N}_{g}}\,{p}_{ij}(g)\\  & = & \mathop{\sum }\limits_{j\mathrm{=2}}^{{N}_{g}}\,{p}_{ij}(g){T}_{j}^{(g)}+{p}_{i1}(g){T}_{1}^{(g)}+1\\  & = & \mathop{\sum }\limits_{j\mathrm{=2}}^{{N}_{g}}\,{p}_{ij}(g){T}_{j}^{(g)}+{p}_{i1}(g)\times 0+1\\  & = & \mathop{\sum }\limits_{j\mathrm{=2}}^{{N}_{g}}\,{p}_{ij}(g){T}_{j}^{(g)}+\mathrm{1,}\end{array}$$

which can be rewritten in matrix form as

2 $$\begin{array}{l}\overline{{{\bf{T}}}_{{\bf{g}}}}=\overline{{{\bf{P}}}_{{\bf{g}}}}\overline{{{\bf{T}}}_{{\bf{g}}}}+\overline{{{\bf{e}}}_{{\bf{g}}}},\end{array}$$

where $\overline{{{\bf{T}}}_{{\bf{g}}}}$ and $\overline{{{\bf{P}}}_{{\bf{g}}}}$ are formed by deleting the first row of **T**_**g**_ and **P**_**g**_ and respectively. The variable ${\bar{{\bf{e}}}}_{{\bf{g}}}$ is formed by removing the first element of **e**_**g**_, which is the *N*_*g*_-dimensional vector of ones.

From Eq. (2), we obtain

3 $$\begin{array}{l}\overline{{{\bf{T}}}_{{\bf{g}}}}=(\overline{{{\bf{I}}}_{{\bf{g}}}}-\overline{{{\bf{P}}}_{{\bf{g}}}}{)}^{-1}\overline{{{\bf{e}}}_{{\bf{g}}}},\end{array}$$

where $\overline{{{\bf{I}}}_{{\bf{g}}}}$ is the (*N*_*g*_ − 1) × (*N*_*g*_ − 1) identity matrix.

From Eq. (3), we further obtain

4 $$\begin{array}{l}{\langle T\rangle }_{g}=\frac{1}{{N}_{g}-1}\mathop{\sum }\limits_{i=2}^{{N}_{g}}\,{T}_{i}^{(g)}=\frac{1}{{N}_{g}-1}\mathop{\sum }\limits_{i=2}^{{N}_{g}}\,\mathop{\sum }\limits_{j=2}^{{N}_{g}}\,{\tau }_{ij}.\end{array}$$

where *τ*_*ij*_ is the *ij*th element of matrix ${(\overline{{{\bf{I}}}_{{\bf{g}}}}-\overline{{{\bf{P}}}_{{\bf{g}}}})}^{-1}$. By definition, 〈*T*〉_*g*_ is the average trapping time (ATT) after *g* iterations. Note that Eq. (4) is valid only if node 1 is designated as absorbing node, or *p*_1,1_(*g*) = 1 and *p*_1,*j*_(*g*) = *p*_*i*,1_(*g*) = 0 for all *i*,*j* > 1.

Equation (4) shows that in order to compute 〈*T*〉_*n*_ or ATT, we only need to compute the inverse of transition matrix. However, computing the inverse of a matrix leads to heavy computational burden when network’s size is large. Therefore, alternative approach is needed to efficiently compute the ATT and equation 4 can be applied to verify the solutions derived by alternative approach.

### Analytical solutions for ATT in weighted directed Koch network

Now, we analytically derive the ATT with a given target node on directed weighted Koch network. Let Ω_*g*_ denote the set of nodes in generation *g* and ${\bar{\Omega }}_{g}$ be the set of new nodes created at generation *g*, then $|{\Omega }_{g}|=|{\Omega }_{g-1}|+|{\bar{\Omega }}_{g}|$. It is obviously that $|{\Omega }_{g}|=2\times {4}^{g}+1$, and $|{\bar{\Omega }}_{g}\mathrm{|\; =\; 6}\times {4}^{g-1}$. Let *k*_*i*_(*g*) represents the degree of node *i* at *gth* generation. According to network’s construction rule, the iteration rule of *k*_*i*_(*g*) is *k*_*i*_(*g*) = 2*k*_*i*_(*g* − 1). Furthermore, among the 2*k*_*i*_(*g* − 1) neighbors of node *i* at generation *g*, there are *k*_*i*_(*g* − 1) neighbors created before generation *g*(old neighbor) and there are *k*_*i*_(*g* − 1) neighbors created just at generation *g*(new neighbor). We use *Z* to denote mean first passage time for a walker moving from node *i* to any of its *k*_*i*_(*g*) old neighbors, and let *X* represent the mean first passage time for a walker going from any of the new neighbors of *i* to one of its *k*_*i*_(*g*) old neighbors. By the iteration rule of the directed weighted Koch network, we have

5 $$\begin{array}{l}\{\begin{array}{rcl}Z & = & \frac{1}{1+\theta }+\frac{\theta }{1+\theta }(1+X),\\ X & = & \frac{1}{1+\theta }(1+Z)+\frac{\theta }{1+\theta }(1+X),\end{array}\end{array}$$

from which we obtain *Z* = (1 + *θ*)^2^, which is just the iteration rule of the average trapping time. In other words,

6 $$\begin{array}{l}{T}_{i}^{(g+1)}={(1+\theta )}^{2}{T}_{i}^{(g)}.\end{array}$$

We define the intermediary quantities for 1 ≤ m ≤ g.

7 $$\begin{array}{l}{T}_{m,tot}^{(g)}=\sum _{i\in {\Omega }_{m}}\,{T}_{i}^{(g)},\end{array}$$

and

8 $$\begin{array}{l}{\bar{T}}_{m,tot}^{(g)}=\sum _{i\in {\bar{\Omega }}_{m}}\,{T}_{i}^{(g)}.\end{array}$$

Then, we obtain the relation

9 $$\begin{array}{l}{\langle T\rangle }_{g}=\frac{1}{{N}_{g}-1}{T}_{g,tot}^{(g)}.\end{array}$$

Thus, the problem of computing 〈*T*〉_*g*_ reduces to computing ${T}_{g,tot}^{(g)}$. Taking the iteration rule of the average trapping time into consideration, we have

10 $$\begin{array}{l}{T}_{g,tot}^{(g)}={T}_{g-1,tot}^{(g)}+{\bar{T}}_{g,tot}^{(g)}={(1+\theta )}^{2}{T}_{g-1,\,tot}^{(g-1)}+{\bar{T}}_{g,tot}^{(g)}.\end{array}$$

Hence, in order to get ${T}_{g,tot}^{(g)}$, we must first obtain ${\bar{T}}_{g,tot}^{(g)}$.

When *g* = 1,

$${T}_{4}^{(1)}=\frac{1}{1+\theta }(1+{T}_{1}^{(1)})+\frac{\theta }{1+\theta }(1+{T}_{5}^{(1)}),$$

$${T}_{5}^{(1)}=\frac{1}{1+\theta }(1+{T}_{1}^{(1)})+\frac{\theta }{1+\theta }(1+{T}_{4}^{(1)}),$$

$${T}_{6}^{(1)}=\frac{1}{1+\theta }(1+{T}_{2}^{(1)})+\frac{\theta }{1+\theta }(1+{T}_{7}^{(1)}),$$

$${T}_{7}^{(1)}=\frac{1}{1+\theta }(1+{T}_{2}^{(1)})+\frac{\theta }{1+\theta }(1+{T}_{6}^{(1)}),$$

$${T}_{8}^{(1)}=\frac{1}{1+\theta }(1+{T}_{3}^{(1)})+\frac{\theta }{1+\theta }(1+{T}_{9}^{(1)}),$$

$${T}_{9}^{(1)}=\frac{1}{1+\theta }(1+{T}_{3}^{(1)})+\frac{\theta }{1+\theta }(1+{T}_{8}^{(1)}).$$

Thus, we have

11 $$\begin{array}{rcl}{\bar{T}}_{\mathrm{1,}tot}^{\mathrm{(1)}} & = & \sum _{i\in {\bar{\Omega }}_{1}}\,{T}_{{\rm{i}}}^{\mathrm{(1)}}\\  & = & {T}_{4}^{(1)}+{T}_{5}^{(1)}+{T}_{6}^{(1)}+{T}_{7}^{(1)}+{T}_{8}^{(1)}+{T}_{9}^{(1)}\\  & = & 6(1+\theta )+2({T}_{1}^{(1)}+{T}_{2}^{(1)}+{T}_{3}^{(1)})\\  & = & 6(1+\theta )+2{\bar{T}}_{0,tot}^{(1)}.\end{array}$$

Similarly, when *g* = 2, we obtain

12 $$\begin{array}{rcl}{\bar{T}}_{\mathrm{2,}tot}^{\mathrm{2)}} & = & \sum _{i\in {\bar{\Omega }}_{2}}\,{T}_{i}^{(2)}=\mathop{\sum }\limits_{i\in {\bar{\Omega }}_{10}}^{33}\,{T}_{i}^{(2)}\\  & = & {T}_{4}^{(1)}+{T}_{5}^{(1)}+{T}_{6}^{(1)}+{T}_{7}^{(1)}+{T}_{8}^{(1)}+{T}_{9}^{(1)}\\  & = & 24(1+\theta )+{2}^{2}({T}_{1}^{(2)}+{T}_{2}^{(2)}+{T}_{3}^{(2)})+2({T}_{4}^{(2)}+{T}_{5}^{(2)}+{T}_{6}^{(2)}+{T}_{7}^{(2)}+{T}_{8}^{(2)}+{T}_{9}^{(2)})\\  & = & 6(1+\theta )\times {4}^{1}+{2}^{2}{\bar{T}}_{0,tot}^{(2)}+2{\bar{T}}_{1,tot}^{(2)}.\end{array}$$

From Eqs (10) and (11), we obtain

13 $$\begin{array}{rcl}{\bar{T}}_{g,tot}^{(g)} & = & 6(1+\theta )\times {4}^{g-1}+2{\bar{T}}_{g-1,tot}^{(g)}+{2}^{2}{\bar{T}}_{g-2,tot}^{(g)}+\mathrm{..}.+{2}^{g-1}{\bar{T}}_{1,tot}^{(g)}+{2}^{g}{\bar{T}}_{0,tot}^{(g)}\\  & = & \mathrm{(1}+\theta )|{\bar{\Omega }}_{g}|+\sum _{{\rm{i}}\in {\Omega }_{g-1}}\,[{k}_{i}(g-\mathrm{1)}\times {T}_{i}^{(g)}],\end{array}$$

and

14 $$\begin{array}{rcl}{\bar{T}}_{g+\mathrm{1,}tot}^{(g+\mathrm{1)}} & = & \mathrm{6(1}+\theta )\times {4}^{g}+2{\bar{T}}_{g,tot}^{(g+\mathrm{1)}}+{2}^{2}{\bar{T}}_{g-\mathrm{1,}tot}^{(g+\mathrm{1)}}+\ldots +{2}^{g}{\bar{T}}_{\mathrm{1,}tot}^{(g)}+{2}^{g+1}{\bar{T}}_{\mathrm{0,}tot}^{(g)}\\  & = & \mathrm{(1}+\theta )|{\bar{\Omega }}_{g+1}|+\sum _{{\rm{i}}\in {\Omega }_{g}}\,[{k}_{i}(g)\times {T}_{i}^{(g+\mathrm{1)}}\mathrm{]}.\end{array}$$

Multiplying Eq. (13) with (1 + *θ*)^2^ and subtracting the result from Eq. (14), we find

15 $${\bar{T}}_{g+1,tot}^{(g+1)}-{(1+\theta )}^{2}{\bar{T}}_{g,tot}^{(g)}=6(1+\theta )\times {4}^{g}-6(1+\theta )\times {4}^{g-1}\times 2{(1+\theta )}^{2}+2{\bar{T}}_{g,tot}^{g+1},$$

which can be rewritten as

16 $$\begin{array}{rcl}{\bar{T}}_{g+1,tot}^{(g+1)} & = & 4{(1+\theta )}^{2}{\bar{T}}_{g,tot}^{(g)}-12(1+\theta )({\theta }^{2}+2\theta -1)\times {4}^{g-1}\\  & = & 4{(1+\theta )}^{2}{\bar{T}}_{g,tot}^{(g)}-3(1+\theta )({\theta }^{2}+2\theta -1)\times {4}^{g}.\end{array}$$

Using ${\bar{T}}_{\mathrm{1,}tot}^{\mathrm{(1)}}=\mathrm{2(1}+\theta \mathrm{)(7}+4\theta )$, Eq. (16) can be resolved inductively to yield

17 $$\begin{array}{rcl}{\bar{T}}_{g,tot}^{(g)} & = & {4}^{g-1}{(1+\theta )}^{2g-1}[2(7+4\theta )-\frac{3({\theta }^{2}+2\theta -1)}{2\theta +{\theta }^{2}}]\\  &  & \,+\,\frac{3({\theta }^{2}+2\theta -1)(\theta +1)\times {4}^{g-1}}{2\theta +{\theta }^{2}}.\end{array}$$

When *θ* = 1, substituting Eq. (17) into Eq. (10), we obtain

18 $$\begin{array}{l}{T}_{g,tot}^{(g)}=\frac{{4}^{g}}{3}(10\times {4}^{g}+3g+2).\end{array}$$

When *θ* = 1, the result is consistent with the relevant expression in literature^39^. From Eq. (18), we find that the ATT grows linearly with the network size. In fact, when *θ* = 1, the network reduces to binary Koch network.

When *θ* ≠ 1, we have

19 $$\begin{array}{rcl}{T}_{g,tot}^{(g)} & = & \,{(1+\theta )}^{2g-1}\times (\frac{{4}^{g}-1}{3})\times [2(7+4\theta )-\frac{3({\theta }^{2}+2\theta -1)}{2\theta +{\theta }^{2}}]\\  &  & +\,\frac{3({\theta }^{2}+2\theta -1)\times (\theta +1)}{2\theta +{\theta }^{2}}[\frac{{4}^{g}-{(1+\theta )}^{2g}}{4-{(1+\theta )}^{2}}]+4{(1+\theta )}^{2g}.\end{array}$$

Thus, according to the Eq. (9), we obtain

20 $$\begin{array}{rcl}{\langle T\rangle }_{g} & = & \frac{1}{{N}_{g}-1}{T}_{g,tot}^{(g)}\\  & = & \,{\mathrm{(1}+\theta )}^{2g-1}\times \frac{1-\frac{1}{{4}^{g}}}{6}\times [\mathrm{2(7}+4\theta )-\frac{\mathrm{3(}{\theta }^{2}+2\theta -\mathrm{1)}}{2\theta +{\theta }^{2}}]\\  &  & +\,\frac{\mathrm{3(}{\theta }^{2}+2\theta -\mathrm{1)}\times (\theta +\mathrm{1)}}{\mathrm{2(2}\theta +{\theta }^{2})}[\frac{1-{(\frac{1+\theta }{2})}^{2g}}{4-{\mathrm{(1}+\theta )}^{2}}]+2{(\frac{1+\theta }{2})}^{2g},\end{array}$$

when *g* → ∞ and *θ* ≠ 1, we have

21 $$\begin{array}{rcl}{\langle T\rangle }_{g} & \approx  & {(1+\theta )}^{2g}[\frac{7+4\theta }{3(\theta +1)}-\frac{{\theta }^{2}+2\theta -1}{2(2\theta +{\theta }^{2})(1+\theta )}]\\  & \approx  & {N}_{g}^{{\log }_{2}(\theta +1)}.\end{array}$$

Equation (21) shows that 〈*T*〉_*g*_ grows sub-linearly with the network size if *θ* < 1, while 〈*T*〉_*g*_ grows super-linearly with the network size if *θ* > 1.

**Figure 3:**
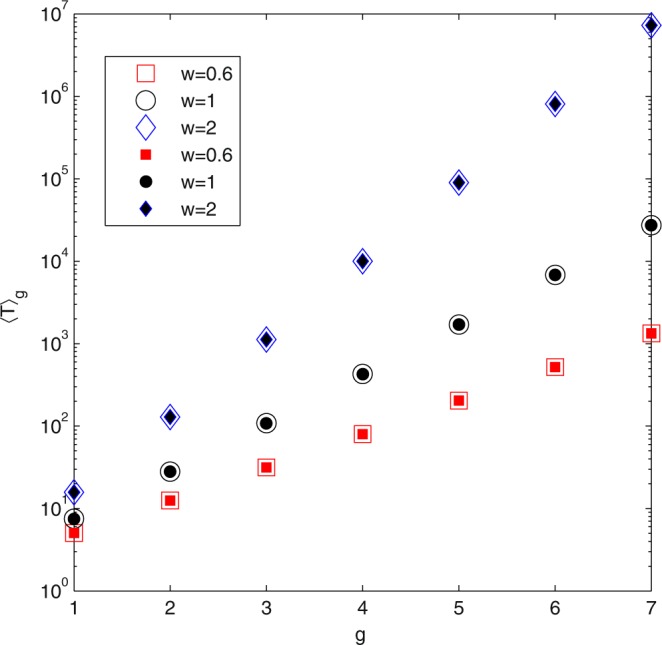
Verification of analytical solutions on weighted Koch network. ATT 〈*T*〉_*g*_ for three different values of *θ* and *g*. The filled symbols represent the data of numerical results and the empty symbols are the analytical values given by Eqs (19) or (20).

We have compared our analytical result with numerical result obtained from Eq. (4). They agree well with each other for different values of *θ* and network order *g*, which are summarized in Fig. 3.

### Expressions for ATT with delay parameter on weighted directed Koch network

We now introduce a delay parameter *p* to explore the impact of time-delay on random walk.

By the construction of network, we define the random walk process in the network with delay as follows: The walker in $\overrightarrow{K}(g-\mathrm{1)}$ is allowed to move to any neighbor either in $\overrightarrow{K}(g-\mathrm{1)}$ or $\overrightarrow{K}(g)$ with probability *p* and 1 − *p*, respectively, where 0 ≤ *p* ≤ 1. The transition probability *p*_*ij*_ for the walker jumping from node *i* to node *j* is defined by

22 $${{\rm{p}}}_{ij}=\{\begin{array}{ll}p\times \frac{{W}_{ij}(g)}{{s}_{i}^{+}(g-\mathrm{1)}}+\mathrm{(1}-p)\times \frac{{W}_{ij}(g)}{{s}_{i}^{+}(g)}, & j\in {\Omega }_{g-1}\\ \mathrm{(1}-p)\times \frac{{W}_{ij}(g)}{{s}_{i}^{+}(g)}. & j\in {\bar{\Omega }}_{g},\end{array}$$

where ${s}_{i}^{+}(g)$ is the out-strength of node *i*. If the walker is at nodes newly created in generation *g*, it will do random walk on $\overrightarrow{K}(g)$.

When *p* = 0, the walker moves from its current location (node *i*) to any neighboring node *j* with transition probability ${p}_{ij}(g)={W}_{ij}(g)/{S}_{i}^{+}(g)$. In other words, random walk with delay reduces to random walk in $\overrightarrow{K}(g)$.

When *p* = 1, the walker jumps from current position (node *i*) to any neighboring node *j* in $\overrightarrow{K}(g-\mathrm{1)}$ with transition probability ${p}_{ij}(g)={W}_{ij}(g)/{S}_{i}^{+}(g-\mathrm{1)}$ if node *i* is created before generation *g*. Otherwise, the walker moves from current position to any neighbor in $\overrightarrow{K}(g)$ with transition probability ${p}_{ij}(g)={W}_{ij}(g)/{S}_{i}^{+}(g)$.

By the definition above, the quantities of *Z* and *X* obeys the relation

23 $$\begin{array}{l}\{\begin{array}{rcl}Z & = & p+(1-p)[\frac{1}{1+\theta }+\frac{\theta }{1+\theta }(1+X)],\\ X & = & \frac{1}{1+\theta }(1+Z)+\frac{\theta }{1+\theta }(1+X),\end{array}\end{array}$$

from which we have $Z=\frac{\mathrm{(1}+\theta -\theta p\mathrm{)(1}+\theta )}{1+\theta p}$.

In this section, we define *D*_*i*_(*g*) as the MFPT in $\overrightarrow{K}(g)$ with the delay state and 〈*D*〉_*g*_ as the average trapping time in $\overrightarrow{K}(g)$. According Eq. (23), we have

24 $$\begin{array}{l}{D}_{i}(g+1)=\frac{(1+\theta -\theta p)(1+\theta )}{1+\theta p}{D}_{i}(g).\end{array}$$

To obtain 〈*D*〉_*g*_, we need to define two quantities ${D}_{m,tot}^{(g)}={\sum }_{{\rm{i}}\in {\Omega }_{m}}{D}_{{\rm{i}}}^{(g)}$ and ${\bar{D}}_{m,tot}^{(g)}={\sum }_{{\rm{i}}\in {\bar{\Omega }}_{m}}{D}_{{\rm{i}}}^{(g)}$, where *m* ≤ *g*. With ${D}_{m,tot}^{(g)}$ and ${\bar{D}}_{m,tot}^{(g)}$ at hand, we have

25 $$\begin{array}{l}{\langle D\rangle }_{g}=\frac{1}{{N}_{g}-1}{D}_{g,tot}^{(g)},\end{array}$$

where

26 $$\begin{array}{rcl}{D}_{g,tot}^{(g)} & = & {D}_{g-1,tot}(g)+{\bar{D}}_{g,tot}(g)\\  & = & \frac{(1+\theta -\theta p)(1+\theta )}{1+\theta p}{T}_{g-1,tot}(g-1)+{\bar{D}}_{g,tot}(g),\end{array}$$

and the ${\bar{D}}_{g,tot}^{(g)}$ can be computed as follows:

27 $$\begin{array}{rcl}{\bar{D}}_{g,tot}^{(g)} & = & (1+\theta )|{\bar{\Omega }}_{g}|+\sum _{i\in {\varOmega }_{g-1}}\,[{k}_{i}(g-1)\times {D}_{i}^{(g)}]\\  & = & (1+\theta )|{\bar{\Omega }}_{g}|+\sum _{i\in {\varOmega }_{g-1}}\,[{k}_{i}(g-1)\times \frac{(1+\theta -\theta p)(1+\theta )}{1+\theta p}{T}_{i}^{(g-1)}],\end{array}$$

and

28 $$\begin{array}{l}{\bar{D}}_{g+1,tot}^{(g+1)}=(1+\theta )|{\bar{\Omega }}_{g+1}|+\sum _{i\in {\Omega }_{g}}\,[{k}_{i}(g-1)\times \frac{(1+\theta -\theta p)(1+\theta )}{1+\theta p}{T}_{i}^{(g)}].\end{array}$$

Multiplying Eq. (26) with 2(*θ* + 1)^2^ and subtracting the result from Eq. (28), we find

29 $$\begin{array}{c}{\bar{D}}_{g+1,tot}^{(g+1)}-2{(\theta +1)}^{2}{\bar{D}}_{g,tot}^{(g)}\\ \,=\,\mathrm{(1}+\theta )[|{\bar{\Omega }}_{g+1}|-\mathrm{2(}\theta +{\mathrm{1)}}^{2}|{\bar{\Omega }}_{g}|]+2\frac{\mathrm{(1}+\theta -\theta p\mathrm{)(1}+\theta )}{\mathrm{(1}+\theta p)}\sum _{{\rm{i}}\in {\bar{\Omega }}_{g}}\,{T}_{{\rm{i}}}^{(g+\mathrm{1)}}\\ \,=\,\mathrm{(1}+\theta )[|{\bar{\Omega }}_{g+1}|-\mathrm{2(}\theta +{\mathrm{1)}}^{2}|{\bar{\Omega }}_{g}|]+2\frac{\mathrm{(1}+\theta -\theta p\mathrm{)(1}+\theta )}{\mathrm{(1}+\theta p)}{\bar{T}}_{g,\text{tot}}^{(g)}.\end{array}$$

Plugging $|{\bar{\Omega }}_{g}|=6\times {4}^{g-1}$ and Eq. (17) into Eq. (29), and taking ${\bar{D}}_{\mathrm{1,}tot}^{\mathrm{(1)}}=\frac{\mathrm{2(1}+\theta \mathrm{)(7}+4\theta -\theta p)}{1+\theta p}$ into consideration, we compute

30 $$\begin{array}{ll}{\bar{D}}_{g+\mathrm{1,}tot}^{(g+\mathrm{1)}} & =\,2(1+\theta {)}^{2}{\bar{D}}_{g,tot}^{(g)}-\mathrm{3(1}+\theta )({\theta }^{2}+2\theta -\mathrm{1)}\times {4}^{g}\\  & \,+\,2\times {4}^{g-1}\times \frac{{\mathrm{(1}+\theta )}^{2g}\mathrm{(1}+\theta -\theta p)}{\mathrm{(1}+\theta p)}\times [\mathrm{2(7}+4\theta )-\frac{\mathrm{3(}{\theta }^{2}+2\theta -\mathrm{1)}}{2\theta +{\theta }^{2}}]\\  & \,+\,\frac{\mathrm{6(}{\theta }^{2}+2\theta -\mathrm{1)(1}+\theta {)}^{2}\mathrm{(1}+\theta -\theta p)\times {4}^{g-1}}{\mathrm{(2}\theta +{\theta }^{2}\mathrm{)(1}+\theta p)}.\end{array}$$

Based on Eq. (30), we further find

31 $$\begin{array}{rcl}{\bar{D}}_{g,tot}^{(g)} & = & \frac{(7+4\theta -\theta p)}{1+\theta p}{2}^{g}{(1+\theta )}^{2g-1}\\  &  & +\frac{(1+\theta -\theta p)}{1+\theta p}[2(7+4\theta )-\frac{3({\theta }^{2}+2\theta -1)}{2\theta +{\theta }^{2}}]\times {2}^{g-1}{(1+\theta )}^{2g-2}({2}^{g-1}-1)\\  &  & +{2}^{g}\times [\frac{3{(1+\theta )}^{2}(1+\theta -\theta p)}{2(2\theta +{\theta }^{2})(1+\theta p)}-3(1+\theta )]\times [{(1+\theta )}^{2g-2}-{2}^{g-1}].\end{array}$$

When *θ* ≠ 1, we recall Eq. (26) to obtain

32 $$\begin{array}{ll}{D}_{g,tot}^{(g)} & =\,\frac{\mathrm{(1}+\theta -\theta p\mathrm{)(1}+\theta )}{1+\theta p}{T}_{g-\mathrm{1,}tot}^{(g-\mathrm{1)}}+{\bar{D}}_{g,tot}^{(g)}\\  & =\,\frac{\mathrm{(1}+\theta -\theta p)}{1+\theta p}\{{\mathrm{(1}+\theta )}^{2g-2}\times \frac{{4}^{g-1}-1}{3}\times [\mathrm{2(7}+4\theta )-\frac{\mathrm{3(}{\theta }^{2}+2\theta -\mathrm{1)}}{2\theta +{\theta }^{2}}]\\  & \,+\frac{\mathrm{3(}{\theta }^{2}+2\theta -\mathrm{1)}\times {(\theta +\mathrm{1)}}^{2}}{2\theta +{\theta }^{2}}[\frac{{4}^{g-1}-{\mathrm{(1}+\theta )}^{2g-2}}{4-{\mathrm{(1}+\theta )}^{2}}]+\mathrm{4(1}+\theta {)}^{2g-1}\}+{\bar{D}}_{g,tot}^{(g)}.\end{array}$$

By substituting Eq. (31) into Eq. (32), we obtain

33 $$\begin{array}{rcl}{D}_{g,tot}^{(g)} & = & \frac{(1+\theta -\theta p)(1+\theta )}{1+\theta p}{T}_{g-1,tot}^{(g-1)}+{\bar{D}}_{g,tot}^{(g)}\\  & = & \frac{(1+\theta -\theta p)}{1+\theta p}\{{(1+\theta )}^{2g-2}\times \frac{{4}^{g-1}-1}{3}\times [2(7+4\theta )-\frac{3({\theta }^{2}+2\theta -1)}{2\theta +{\theta }^{2}}]\\  &  & +\,\frac{3({\theta }^{2}+2\theta -1)\times {(\theta +1)}^{2}}{2\theta +{\theta }^{2}}[\frac{{4}^{g-1}-{(1+\theta )}^{2g-2}}{4-{(1+\theta )}^{2}}]+4{(1+\theta )}^{2g-1}\}\\  &  & +\,\frac{(7+4\theta -\theta p)}{1+\theta p}{2}^{g}{(1+\theta )}^{2g-1}\\  &  & +\,\frac{(1+\theta -\theta p)}{1+\theta p}[2(7+4\theta )-\frac{3({\theta }^{2}+2\theta -1)}{2\theta +{\theta }^{2}}]\times {2}^{g-1}{(1+\theta )}^{2g-2}({2}^{g-1}-1)\\  &  & +\,{2}^{g}\times [\frac{3{(1+\theta )}^{2}(1+\theta -\theta p)}{2(2\theta +{\theta }^{2})(1+\theta p)}]\times [{(1+\theta )}^{2g-2}-{2}^{g-1}].\end{array}$$

Then from Eq. (25), we have

34 $$\begin{array}{rcl}{\langle D\rangle }_{g} & = & \frac{{D}_{g,tot}^{(g)}}{{N}_{g}-1}\\  & = & \,\frac{\mathrm{(1}+\theta -\theta p)}{\mathrm{2(1}+\theta p)}\{{\mathrm{(1}+\theta )}^{2g-2}\times \frac{1-\frac{1}{{4}^{g-1}}}{12}\times [\mathrm{2(7}+4\theta )-\frac{\mathrm{3(}{\theta }^{2}+2\theta -\mathrm{1)}}{2\theta +{\theta }^{2}}]\\  &  & +\,\frac{\mathrm{3(}{\theta }^{2}+2\theta -\mathrm{1)}\times {(\theta +\mathrm{1)}}^{2}}{2\theta +{\theta }^{2}}[\frac{\frac{1}{4}-{(\frac{1+\theta }{2})}^{2g}}{4-{\mathrm{(1}+\theta )}^{2}}]+\frac{4}{\mathrm{(1}+\theta )}{(\frac{1+\theta }{2})}^{2g}\}\\  &  & +\,\frac{\mathrm{(7}+4\theta -\theta p)}{\mathrm{(1}+\theta p\mathrm{)(1}+\theta )}{(\frac{1+\theta }{2})}^{2g}\\  &  & +\,\frac{\mathrm{(1}+\theta -\theta p)}{\mathrm{2(1}+\theta p\mathrm{)(1}+\theta {)}^{2}}[\mathrm{2(7}+4\theta )-\frac{\mathrm{3(}{\theta }^{2}+2\theta -\mathrm{1)}}{2\theta +{\theta }^{2}}]\times \frac{{\mathrm{(1}+\theta )}^{2g}}{{2}^{g}}{\mathrm{(2}}^{g-1}-\mathrm{1)}\\  &  & +\,[\frac{\mathrm{3(1}+\theta {)}^{2}\mathrm{(1}+\theta -\theta p)}{\mathrm{2(2}\theta +{\theta }^{2}\mathrm{)(1}+\theta p)}-\mathrm{3(1}+\theta )]\,[{[\frac{{\mathrm{(1}+\theta )}^{2}}{2}]}^{g}-\frac{{\mathrm{(1}+\theta )}^{2}}{2}].\end{array}$$

Then, for *g* → ∞, we find

35 $$\begin{array}{rcl}{\langle D\rangle }_{g} & \approx  & \frac{\mathrm{(1}+\theta -\theta p)}{\mathrm{24(1}+\theta p)}[\mathrm{2(7}+4\theta )-\frac{\mathrm{3(}{\theta }^{2}+2\theta -\mathrm{1)}}{2\theta +{\theta }^{2}}]{\mathrm{(1}+\theta )}^{2g-2}\\  & \approx  & \frac{\mathrm{(1}+\theta -\theta p)}{\mathrm{24(1}+\theta p)}[\mathrm{2(7}+4\theta )-\frac{\mathrm{3(}{\theta }^{2}+2\theta -\mathrm{1)}}{2\theta +{\theta }^{2}}]{N}_{g}^{lo{g}_{2}(\theta +\mathrm{1)}}.\end{array}$$

When *θ* = 1, $Z=\frac{\mathrm{2(2}-p)}{\mathrm{(1}+p)}$, we have

36 $$\begin{array}{l}{D}_{i}(g+1)=\frac{2(2-p)}{1+p}{T}_{i}(g).\end{array}$$

Plugging *θ* = 1 into (31), we have

37 $$\begin{array}{rcl}{\bar{D}}_{g,tot}^{(g)} & = & \,\frac{4(11-p)}{1+p}\times {2}^{3g-3}+\frac{5(2-p)}{1+p}\times {2}^{3g-1}({2}^{g-1}-1)\\  &  & +\,[\frac{2-p}{1+p}-3]\times {2}^{2g}({2}^{g-1}-1).\end{array}$$

Therefore, we obtain

38 $$\begin{array}{ll}{D}_{g,tot}^{(g)} & =\,\frac{\mathrm{(2}-p)}{\mathrm{6(1}+p)}\times {4}^{g}\mathrm{(10}\times {4}^{g-1}+3g-\mathrm{1)}+\frac{\mathrm{4(11}-p)}{1+p}\times {2}^{3g-3}\\  & \,+\,5\frac{2-p}{1+p}\times {2}^{3g-1}{\mathrm{(2}}^{g-1}-\mathrm{1)}+[\frac{2-p}{1+p}-3]\times {2}^{2g}{\mathrm{(2}}^{g-1}-\mathrm{1)}.\end{array}$$

Thus,

39 $$\begin{array}{rcl}{\langle D\rangle }_{g} & = & \frac{{D}_{g,tot}^{(g)}}{{N}_{g}-1}\\  & = & \frac{\mathrm{(2}-p)}{\mathrm{12(1}+p)}\mathrm{(10}\times {4}^{g-1}+3g-\mathrm{1)}+\frac{\mathrm{(11}-p)}{1+p}\times {2}^{g-2}\\  &  & +\,5\frac{2-p}{1+p}\times {2}^{g-2}{\mathrm{(2}}^{g-1}-\mathrm{1)}+[\frac{2-p}{\mathrm{2(1}+p)}-\frac{3}{2}]\times {\mathrm{(2}}^{g-1}-\mathrm{1)}\\  & \approx  & \frac{\mathrm{35(2}-p)}{\mathrm{24(1}+p)}{N}_{g}.\end{array}$$

**Figure 4:**
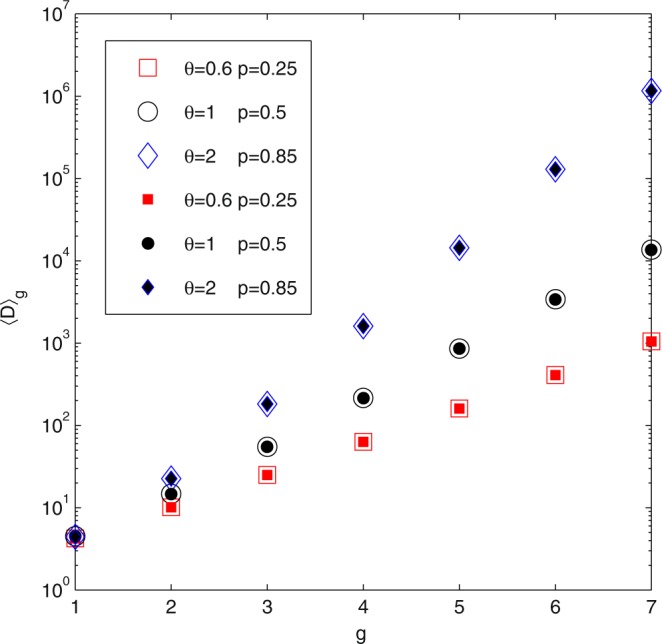
Verification of analytical solutions on weighted Koch network in delay state. ATT 〈*D*〉_*g*_ for weighted directed Koch network in delay state with different *p*, *θ* and *g*. The filled symbols are the results of numerical results of Eq. (4) and the empty symbols represent the exact analytical values of Eqs (34) or (38).

We compared analytical expressions in Eqs (34) and (38) with numerical results from Eq. (4). As shown in Fig. 4, the analytical results agree well with numerical results for different values of *θ*, *p* and n.

It can be seen from Eqs (35) and (39) that 〈*D*〉_*g*_ grows sub-linearly, linearly, super-linearly with network size when *θ* < 1, *θ* = 1, *θ* > 1 respectively. However, Eqs (35) and (39) also show that the delay parameter *p* can only change the coefficient of the ATT. Although the delay parameter *p* has less pronounced influence on the transport efficiency, both it and the weight parameter are needed to tune for getting desired average trapping time.

## Discussion

In this paper, we first proposed a weighted directed Koch network, in which the weight of edge is controlled by a parameter *θ*. Then, we derived the closed form solution of average trapping time (ATT) of a random walk with a given trap node. The solution shows that the weighted directed construction has a significant effect on trapping efficiency and the leading scale of ATT can be sub-linear, linear and super-linear with varying weight parameter. Finally, when a delay is introduced such that new extended structures do not always affect the system immediately, then we introduce a delay parameter *p* to steer the random walk on the weighted directed network. The exact solution of the ATT in this scenario shows that the delay parameter can only change the prefactor of the ATT. Our results also show that we can obtain desired ATT with appropriate values of weight parameter *θ* and delay parameter *p*. It is noteworthy that the procedure used here is limited to deriving average trapping time of random walks with trap located at node 1,2 or 3. For next steps, we will generalize our work to average trapping time with trap located at any nodes.
